# Cirrhosis and Advanced Fibrosis in Hispanics in Texas: The Dominant Contribution of Central Obesity

**DOI:** 10.1371/journal.pone.0150978

**Published:** 2016-03-07

**Authors:** Jingjing Jiao, Gordon P. Watt, MinJae Lee, Mohammad H. Rahbar, Kristina P. Vatcheva, Jen-Jung Pan, Joseph B. McCormick, Susan P. Fisher-Hoch, Michael B. Fallon, Laura Beretta

**Affiliations:** 1 Department of Molecular and Cellular Oncology, University of Texas MD Anderson Cancer Center, Houston, Texas, United States of America; 2 School of Public Health, University of Texas Health Science Center at Houston, Brownsville Regional Campus, Brownsville, Texas, United States of America; 3 Department of Internal Medicine, Division of Clinical and Translational Sciences, The University of Texas Medical School at Houston, Houston, Texas, United States of America; 4 Biostatistics/Epidemiology/Research Design (BERD) Core, Center for Clinical and Translational Sciences, The University of Texas Health Science Center at Houston, Houston, Texas, United States of America; 5 The University of Texas School of Public Health at Houston, Houston, Texas, United States of America; 6 Department of Internal Medicine, Division of Gastroenterology, Hepatology and Nutrition, The University of Texas Medical School at Houston, Houston, Texas, United States of America; Drexel University College of Medicine, UNITED STATES

## Abstract

Liver cirrhosis is a leading cause of death in Hispanics and Hispanics who live in South Texas have the highest incidence of liver cancer in the United States. We aimed at determining the prevalence and associated risk factors of cirrhosis in this population. Clinical and demographic variables were extracted for 2466 participants in the community-based Cameron County Hispanic Cohort in South Texas. Aspartate transaminase to Platelet Ratio Index (APRI) was used to predict cirrhosis in Cameron County Hispanic Cohort. The prevalence of cirrhosis using APRI≥2 was 0.94%, which is nearly 4-fold higher than the national prevalence. Using APRI≥1, the overall prevalence of cirrhosis/advanced fibrosis was 3.54%. In both analyses, highest prevalence was observed in males, specifically in the 25–34 age group. Risk factors independently associated with APRI≥2 and APRI≥1 included hepatitis C, diabetes and central obesity with a remarkable population attributable fraction of 52.5% and 65.3% from central obesity, respectively. Excess alcohol consumption was also independently associated with APRI≥2. The presence of *patatin-like phospholipase domain-containing-3* gene variants was independently associated with APRI≥1 in participants >50 years old. Males with both central obesity and excess alcohol consumption presented with cirrhosis/advanced fibrosis at a young age. Alarmingly high prevalence of cirrhosis and advanced fibrosis was identified in Hispanics in South Texas, affecting young males in particular. Central obesity was identified as the major risk factor. Public health efforts are urgently needed to increase awareness and diagnosis of advanced liver fibrosis in Hispanics.

## Introduction

Liver fibrosis is a wound-healing response characterized by the accumulation of extracellular matrix (ECM) following liver injury such as hepatitis C, hepatitis B, excess alcohol consumption and nonalcoholic fatty liver disease (NAFLD) [1]. When the injury is sustained, chronic inflammation and accumulation of ECM persist, leading to a progressive substitution of liver parenchyma by scar tissue. This process may result in cirrhosis, the end stage of progressive fibrosis, which is associated with poor outcome and high mortality [2]. Liver cirrhosis is a significant health burden, accounting for 49,538 deaths in the United States (US) in 2010 [3]. Liver cirrhosis is the most common risk factor for hepatocellular carcinoma (HCC), a cancer with few curative treatment options and poor overall survival [4].

The prevalence of cirrhosis worldwide is largely unknown [5].A recent study estimated the prevalence of cirrhosis in the US to be 0.27% [6]. In a European study among individuals ≥45 years old, the prevalence of advanced fibrosis or cirrhosis was estimated at 0.6% [7]. Ethnic differences in the incidence of cirrhosis and progression rates have been observed [8]. In the US, chronic liver disease and cirrhosis is the 6^th^ leading cause of death in Hispanics while it is not among the 10 leading causes of death for non-Hispanic whites (NHW) and African Americans [9]. A more aggressive pattern of disease and worse treatment outcome for chronic liver diseases have also been reported in Hispanics [10]. NAFLD is also more common in Hispanics than in NHW or African Americans and is the most prevalent chronic liver disease in Hispanics [11–13].Lifestyle, environmental and genetic risk factors are likely responsible for the ethnic variation in NAFLD prevalence. One such genetic factor is the patatin-like phospholipase domain-containing-3 (PNPLA3) gene. PNPLA3 single-nucleotide polymorphisms (SNPs) rs738409 and rs2281135 are strongly associated with hepatic fat content [14–17] and elevated liver aminotransferase levels [18, 19]. An association between PNPLA3 rs738409 and cirrhosis or advanced fibrosis was also reported in patients with NAFLD [20], alcoholic liver disease [21, 22] or chronic hepatitis C [23, 24]. The association with cirrhosis has been confirmed using meta-analysis [25]. In this study, we aimed to determine the prevalence and associated clinical features of cirrhosis and advanced fibrosis in Hispanics in South Texas. To that end, we interrogated a community-based Hispanic cohort in the US-Mexico Border region, the Cameron County Hispanic Cohort (CCHC). The CCHC is a ‘Framingham-like’ cohort of a Mexican-American community, recruited from households, that was initiated in 2004 [26]. The prevalence of obesity (51%), diabetes (28%) and elevated liver enzymes (39%) is particularly high in this community [26–28].

## Materials and Methods

### Data Sources and Subject Parameters

The Cameron County Hispanic Cohort (CCHC) is the data source for this analysis [26]. Our analysis was performed on CCHC data collected from Jan 29, 2004 to April 18, 2015 and included 2466 participants from the cities of Brownsville and Harlingen, Texas. The study was approved by the Committee for the Protection of Human Subjects of the University of Texas Health Science Center at Houston as HSC-SPH-03-007-B. Written informed consent was given by participants for their clinical records to be used in this study. Aspartate transaminase (AST) to Platelet Ratio Index (APRI) score was calculated as follows: (AST/upper-limit of normal)/platelet count X 100. Upper-limit of normal AST used for calculation was 33IU/L [29]. When participants have APRI scores from multiple visits, data from the last visit were used for the analysis. Diabetes was defined as fasting blood glucose ≥126mg/dl or history of use of diabetic medication. Obesity was defined as a body mass index (BMI) ≥30kg/m^2^. Central obesity was defined as waist circumference ≥ 102cm for men and ≥ 88cm for women. We defined excess alcohol consumption as >2 drinks/d for men and >1 drink/d for women within 1 year before the completion of data collection [30]. A total of 961 participants were tested for hepatitis C and were defined as positive if they had a positive test for antibody to hepatitis C as measured using the ORTHO® HCV Version 3.0 Test system (Ortho-Clinical Diagnostics, Raritan, NJ). All other laboratory tests reported were measured at a CLIA approved community reference laboratory except for insulin, which was measured in the laboratory using Elisa assays (Mercodia®, Uppsala, Sweden). PNPLA3 rs2281135 and rs738409 SNPs were genotyped in 1090 participants by TaqMan 5'-nuclease assays using two predesigned TaqMan probes (Applied Biosystems, Foster City, CA, C_7241_10 for rs738409 and C_15875080_10 for rs2281135). Genotyping was performed on a ViiA7 Real time PCR system (Applied Biosystems, Foster City, CA).

### Statistical Analysis

The analysis took into account the study design and used age- and gender-adjusted sampling weights to scale the sample to the underlying population. In the analysis, we also accounted for clustering effect among participants from the same household. Categorical variables for demographic and clinical characteristics were summarized in weighted percentages, and continuous variables were summarized using weighted means, standard errors and medians. The Rao-Scott design-adjusted chi-square test was used to test for equality of proportions of categorical variables between the cirrhotic and non-cirrhotic participants. Univariable survey-weighted logistic regression analyses were performed to assess the effects of continuous variables on cirrhosis status. Multivariable weighted data analyses were conducted using mixed effect logistic regression models that account for correlations among survey-weighted observations within/between multiple cluster levels. Multicollinearity and interaction effects between the independent variables and potential confounders were examined while developing multivariable models. ANOVA was used to test association between APRI score and 2 PNPLA3 SNPs (rs2281135 and rs738409). Multiple pairwise comparisons were evaluated by Tukey's test. PNPLA3 SNPs rs2281135 and rs738409 were highly correlated (r = 0.91) and separate survey-weighted logistic regression models were created using rs738409. Each SNP was considered as categorical variables with 3 categories (WT allele, 1 risk allele and 2 risk alleles). Population Attributable fraction (PAF) is an estimate of the burden of the disease in the population that could be avoided if the exposure to a specific risk factor was reduced or eliminated. Based on the final multivariable weighted logistic regression models adjusted attributable fractions were derived for each of the factors of interest included in the models. The category-specific attributable risk fraction was estimated by ${PAF}_{i}=\frac{p_{i}({OR}_{i}-1)}{{OR}_{i}}x100\%$ where OR_i_ is the i-th category specific weighted adjusted odds ratio for cirrhosis calculated based on the logistic regression model and p_i_ is the weighted proportion of all cases of cirrhosis falling into i-th exposure level of a categorical variable with k levels and reference level denoted by i = 1. The category-specific attributable risk factor for the unexposed group (i = 1) is 0 since OR_i_ is 1 by definition. The total population attributable risk fraction for a categorical variable with k levels was estimated by $PAF=\sum_{i=2}^{k}{PAF}_{i}$. A two-sided test with P<0.05 was considered significant for all analyses. All statistical analyses were conducted using SAS 9.4 (SAS Institute, Inc., Cary, North Carolina).

## Results

### Prevalence of Cirrhosis in Hispanics in Cameron County

**Fig 1:**
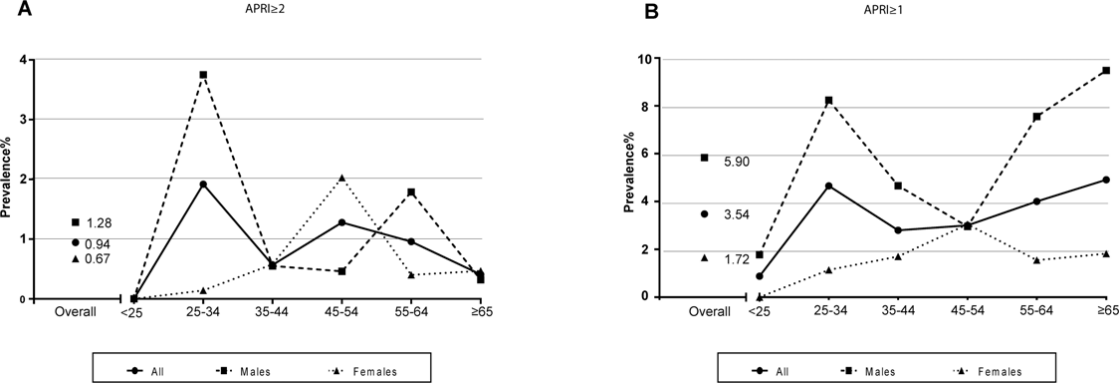
Prevalence of cirrhosis (A) and of advanced fibrosis (B) by age group.

The prevalence of cirrhosis in Hispanics in Cameron County was determined using the Aspartate transaminase (AST) to Platelet Ratio Index (APRI) score ≥2. Using this approach, the overall prevalence of cirrhosis in Cameron County was estimated as 0.94% (Fig 1A). High prevalence (1.28%) was observed in the 45–54 age group in our study group. However, the highest prevalence (1.92%) was observed in the 25–34 age group. This high prevalence in the young age group was mostly contributed by males (Fig 1A).

### Demographic, Clinical and Laboratory Variables by Cirrhosis Status

The demographic, clinical and laboratory features of the participants with cirrhosis (Table 1) included higher levels of liver enzymes, insulin and glucose and significantly lower levels of high density lipoprotein (HDL). Cirrhosis also affected Hispanic females in South Texas. Cirrhosis was also present in a younger age group in Hispanics in the CCHC (mean = 45 years). Among the known risk factors associated with cirrhosis, 11.4% of the participants with cirrhosis reported excess alcohol consumption and 14.2% had hepatitis C.

**Table 1:** Demographic, clinical, and laboratory variables by cirrhosis status (APRI 2 cutoff).

Variables	APRI≥2 (N = 23)	APRI<2 (N = 2443)	*P*
**Age (y)**	45.0(3.8)-*42*.*3*	47.4(0.7)-*46*.*8*	0.53
**Gender**			0.25
Male	59.6%	43.5%	
female	40.4%	56.5%	
**Diabetes**	42.2%	21.6%	0.09
**Excess alcohol consumption**	11.4%	4.5%	0.13
**Blood tests**			
AST	150.7(21.7)-*123*.*2*	29.1(0.6)-*24*.*6*	< .001
ALT	119.1(29.7)-*71*.*3*	36.3(0.8)-*30*.*7*	< .001
ALP	111.9(25.6)-*81*.*6*	67.2(2.0)-*75*.*1*	< .001
Platelets	133.2(21.8)-*140*.*8*	244.1(2.1)-*237*.*2*	< .001
total bilirubin	1.1(0.4)-*0*.*5*	0.5(0.1)-*0*.*4*	0.10
Creatinine	0.9(0.1)-*0*.*7*	0.8(0.01)-*0*.*7*	0.19
HCV antibody	14.2%	2.2%	< .001
HBV surface antigen	5.3%	1.6%	0.22
fasting glucose	144.4(28.2)-*101*.*1*	110.2(1.6)-*96*.*4*	0.04
fasting insulin	19.8(2.9)-*14*.*5*	14.7(0.3)-*11*.*6*	0.008
fasting triglycerides	196.9(46.2)-*120*.*7*	152.4(4.2)-*123*.*9*	0.11
HDL	37.7(2.9)-*37*.*5*	46.3(0.5)-*44*.*6*	0.01
LDL	94.5(13.2)-*108*.*2*	104.7(1.2)-*101*.*8*	0.48
insulin resistance (HOMA)	9.0(2.1)-*5*.*3*	4.1(0.1)-*3*.*0*	0.08
**BMI**	30.0(1.2)-*29*.*3*	31.1(0.3)-*30*.*3*	0.38
**waist circumference**	109.9(5.8)-*103*.*1*	104.2(0.7)-*102*.*9*	0.28
**systolic blood pressure**	121.5(3.9)-*123*.*9*	119.0(0.7)-*115*.*9*	0.49
**diastolic blood pressure**	76.0(2.0)-*74*.*4*	73.7(0.4)-*73*.*2*	0.25
**metabolic syndrome components**			
*waist circumference (cm) (>102/men—>88/women)*	69.0%	73.0%	0.75
*fasting plasma glucose (>110mg/dL)*	42.6%	23.9%	0.14
*blood pressure (>130/85mm Hg)*	32.1%	23.1%	0.49
*Triglycerides (>150mg/dL)*	43.0%	36.0%	0.63
*low HDL (mg/dL) (<40/men—<50/women)*	60.5%	49.9%	0.45

Data are presented as mean (SE)-*median* or as frequency (%).

AST, aspartate aminotransferase; ALT, alanine aminotransferase; ALP, alkaline phosphatase; HCV, hepatitis C virus; HBV, hepatitis B virus; HDL, high-density lipoprotein; LDL, low-density lipoprotein; HOMA, Homeostasis Model Assessment; BMI, body mass index.

### Prevalence of Cirrhosis or Advanced Fibrosis in Hispanics in Cameron County

Two large meta-analyses have reported that using APRI≥2, the sensitivity and specificity to detect cirrhosis are 31–46% and 89–91%, whereas when using APRI≥1, the sensitivity increases to 66–76% with a slight decrease in specificity [31, 32]. Using APRI≥1 will also identify some subjects with advanced fibrosis. We therefore expanded our analysis to cirrhosis/advanced fibrosis in Hispanics in Cameron County by using APRI≥1. The overall prevalence of cirrhosis/advanced fibrosis was 3.54% (Fig 1B). While the highest prevalence was observed in participants older than 65 years old (4.91%), high prevalence was also observed in the 25–34 age group (4.65%). With the exception of the 45–54 age group in which the prevalence was 3.05% in females, the prevalence was significantly higher in males than in females in all age groups. The overall prevalence was 5.9% in males and 1.72% in females. The demographic, clinical and laboratory features of the participants with cirrhosis/advanced fibrosis are presented in Table 2. Participants with cirrhosis/advanced fibrosis were more likely to be male, to have diabetes, to present with higher BMI, waist circumference, fasting triglyceride, glucose and insulin levels. Participants with cirrhosis/advanced fibrosis were also most likely to have hepatitis C (7.3%). Females with cirrhosis/advanced fibrosis had higher HCV prevalence (11.5%) and presented with significantly higher bilirubin levels (Table 2). In contrast, hyperlipidemia was observed only in males (Table 2).

**Table 2:** Demographic, Clinical and Laboratory Variables by Cirrhosis/Advanced Fibrosis Status (APRI 1 cutoff).

**Variables**	All			Males			Females		
	APRI≥1(N = 102)	APRI<1(N = 2364)	*P*	APRI≥1(N = 56)	APRI<1(N = 796)	*P*	APRI≥1(N = 46)	APRI<1(N = 1568)	*P*
**Age**	50.6(3.6)-*49*.*7*	47.3(0.7)-*46*.*7*	0.34	50.8(4.9)-*51*.*0*	46.0(0.9)-*46*.*5*	0.33	50.1(2.1)-*46*.*4*	48.2(0.9)-*46*.*9*	0.34
**Gender**			< .001						
Male	72.7%	42.6%							
Female	27.3%	57.4%							
**Diabetes**	42.6%	21.0%	0.004	41.1%	21.1%	0.04	46.4%	21.0%	0.003
**Excess alcohol consumption**	8.7%	4.4%	0.09	12.0%	8.5%	0.43	0%	1.3%	N.A
**Blood tests**									
AST	95.0(9.2)-*75*.*9*	27.8(0.5)-*24*.*0*	< .001	93.3(11.4)-*62*.*3*	30.2(0.7)-*27*.*8*	< .001	99.5(13.4)-*86*.*0*	26.1(0.6)-*21*.*7*	< .001
ALT	93.5(9.8)-*65*.*6*	35.0(0.7)-*30*.*4*	< .001	96.6(12.9)-*65*.*2*	40.4(1.1)-*35*.*6*	< .001	85.1(11.4)-*78*.*5*	31.1(0.9)-*27*.*0*	< .001
ALP	70.4(12.7)-*72*.*2*	67.5(2.0)-*75*.*2*	0.82	69.9(15.0)-*68*.*4*	68.7(2.9)-*75*.*6*	0.90	71.7(19.5)-*16*.*5*	67.1(2.3)-*74*.*1*	0.82
Platelets	165.1(9.4)-*157*.*1*	245.9(2.1)-*238*.*3*	< .001	170.1(12.4)-*159*.*6*	228.9(2.7)-*220*.*5*	< .001	151.6(8.1)-*142*.*8*	258.5(2.9)-*253*.*8*	< .001
total bilirubin	0.7(0.1)-*0*.*5*	0.5(0.1)-*0*.*4*	0.42	0.6(0.04)-*0*.*5*	0.7(0.2)-*0*.*4*	0.34	1.0(0.4)-*0*.*4*	0.4(0.01)-*0*.*3*	0.003
Creatinine	0.9(0.05)-*0*.*8*	0.8 (0.01)-*0*.*71*	0.19	1.0(0.06)-*0*.*9*	0.9(0.02)-*0*.*9*	0.43	0.7(0.04)-*0*.*6*	0.7(0.02)-*0*.*6*	0.92
HCV antibody	7.3%	1.8%	0.004	5.8%	2.9%	0.33	11.5%	0.9%	< .001
HBV surface antigen	2.9%	1.6%	0.39	1.8%	0.6%	0.38	5.3%	2.1%	0.34
fasting glucose	133.2(8.9)-*109*.*2*	109.7(1.6)-*96*.*1*	< .001	132.2(10.0)-*109*.*4*	113.8(2.7)-*97*.*9*	0.03	135.8(19.6)-*105*.*7*	106.6(1.8)-*94*.*3*	0.02
fasting insulin	23.0(2.4)-*20*.*6*	14.3(0.3)-*11*.*4*	0.001	24.8(3.1)-*21*.*1*	14.6(0.5)-*11*.*3*	0.02	18.3(2.5)-*13*.*1*	14.1(0.4)-*11*.*5*	0.03
fasting triglycerides	192.8(17.1)-*166*.*1*	151.4(4.2)-*123*.*4*	0.004	213.4(19.6)-*167*.*6*	165.0(7.3)-*125*.*9*	0.01	136.6(16.8)-*87*.*0*	141.3(4.2)-*121*.*7*	0.81
HDL	41.1(2.2)-*38*.*2*	46.4(0.5)-*44*.*8*	0.04	40.7(3.0)-*37*.*9*	43.6(0.8)-*41*.*2*	0.41	42.2(1.7)-*38*.*3*	48.5(0.6)-*47*.*1*	0.002
LDL	100.2(6.0)-*106*.*3*	104.8(1.2)-*101*.*6*	0.46	101.3(8.2)-*105*.*4*	104.1(1.8)-*102*.*0*	0.74	97.6(5.3)-*99*.*9*	105.3(1.5)-*101*.*5*	0.19
insulin resistance (HOMA)	8.0(1.0)-*5*.*8*	3.9(0.1)-*2*.*9*	< .001	8.0(1.2)-*5*.*7*	4.1(0.2)-*2*.*9*	0.002	7.9(1.8)-*3*.*6*	3.8(0.1)-*2*.*9*	< .001
**BMI**	33.8(1.0)-*33*.*8*	31.0(0.3)-*30*.*2*	0.002	33.9(1.3)-*33*.*9*	31.5(0.5)-*30*.*3*	0.07	33.8(1.4)-*31*.*3*	30.7(0.30)-*29*.*8*	0.008
**waist circumference**	113.4(3.0)-*110*.*1*	103.9(0.7)-*102*.*6*	< .001	113.1(3.7)-*110*.*2*	106.6(1.2)-*104*.*4*	0.08	114.2(4.8)-*106*.*1*	101.9(0.7)-*101*.*1*	0.003
**systolic blood pressure**	121.8(4.9)-*118*.*9*	118.9(0.7)-*115*.*8*	0.53	123.5(6.5)-*119*.*5*	122.1(0.9)-*119*.*9*	0.83	117.3(3.2)-*117*.*3*	116.6(0.8)-*112*.*1*	0.82
**diastolic blood pressure**	72.9(1.5)-*71*.*0*	73.7(0.4)-*73*.*3*	0.58	73.0(1.9)-*70*.*9*	76.4(0.6)-*75*.*6*	0.08	72.6(2.0)-*72*.*7*	71.7(0.4)-*71*.*7*	0.66
**metabolic syndrome components**									
*waist circumference (cm) (>102/men—>88/women)*	80.2%	72.7%	0.22	73.5%	59.1%	0.12	98.0%	82.9%	< .001
*fasting plasma glucose (>110mg/dL)*	45.1%	23.3%	0.006	44.9%	26.7%	0.09	45.6%	20.7%	0.004
*blood pressure (>130/85mmHg)*	34.8%	22.7%	0.17	37.1%	28.1%	0.45	28.6%	18.8%	0.33
*Triglycerides (>150mg/dL)*	55.3%	35.4%	0.01	65.1%	38.3%	0.005	28.6%	33.2%	0.59
*low HDL (mg/dL)(<40/men–<50/women)*	56.3%	49.7%	0.46	50.1%	40.1%	0.39	72.8%	56.9%	0.09

Data are presented as mean (SE)-*median* or as frequency %.Data are presented for all participants and stratified by gender.

AST, aspartate aminotransferase; ALT, alanine aminotransferase; ALP, alkaline phosphatase; HCV, hepatitis C virus; HBV, hepatitis B virus; HDL, high-density lipoprotein; LDL, low-density lipoprotein; HOMA, Homeostasis Model Assessment; BMI, body mass index.

### Factors Associated with Cirrhosis and Advanced Fibrosis based on Multivariable Logistic Regression Analysis

To determine the factors independently associated with cirrhosis (APRI≥2) or cirrhosis/advanced fibrosis (APRI≥1), multivariable logistic regression analysis was performed. Hepatitis C (adjusted Odds Ratio (OR) = 23.47 (95% CI = 3.14–175.41) p = 0.002), diabetes (adjusted OR = 6.19 (95% CI = 0.96–39.85) p = 0.06), excess alcohol consumption (adjusted OR = 3.98 (95% CI = 0.95–16.64) p = 0.06) and central obesity (adjusted OR = 4.18 (95% CI = 0.43–40.59) p = 0.22) were associated with cirrhosis (Table 3). In the context of cirrhosis/advanced fibrosis, independent risk factors were hepatitis C (adjusted OR = 53.70 (95% CI = 4.97–580.48) p = 0.001), diabetes (adjusted OR = 5.09 (95% CI = 1.77–14.67) p = 0.003), central obesity (adjusted OR = 5.40 (95% CI = 1.11–26.33) p = 0.04) and male gender (adjusted OR = 8.03 (95% CI = 2.48–26.01) p < .001) (Table 3). Excess alcohol consumption was not found as a statistically significant risk factor for cirrhosis/advanced fibrosis in this population. BMI was also not found as an independent risk factor for cirrhosis nor advanced fibrosis. Approximately 35.4% of cirrhosis and 34.4% of cirrhosis/advanced fibrosis could be attributed to diabetes (Table 3). Remarkably, 52.5% of cirrhosis and 65.3% of cirrhosis/advanced fibrosis could be attributed to central obesity (Table 3).

**Table 3:** Factors Associated with cirrhosis (APRI≥2) and cirrhosis/advanced fibrosis (APRI≥1) based on multivariable logistic regression models.

**A Cirrhosis model**				
**Variables**	**Adjusted** ^a^ **Odds Ratio**	**95% CI**	***P***	**PAF**
Hepatitis C	23.47	3.14–175.41	0.002	13.6%
Diabetes	6.19	0.96–39.85	0.06	35.4%
Excess alcohol consumption	3.98	0.95–16.64	0.06	8.5%
Central obesity	4.18	0.43–40.59	0.22	52.5%
Male	4.30	0.45–40.72	0.20	
**B Cirrhosis/advanced fibrosis model**				
**Variables**	**Adjusted** ^**a**^ **Odds Ratio**	**95% CI**	***P***	**PAF**
Hepatitis C	53.70	4.97–580.48	0.001	7.2%
Diabetes	5.09	1.77–14.67	0.003	34.4%
Excess alcohol consumption	1.31	0.21–8.09	0.77	2.0%
Central obesity	5.40	1.11–26.33	0.04	65.3%
Male	8.03	2.48–26.01	< .001	

PAF, population attributable fraction; CI, confidence interval. Analysis is based on n = 725 participants.

^a^ Lipidemia and total bilirubin were also adjusted in this multivariable model (not listed)

### *Patatin-like phospholipase domain-containing-3* (PNPLA3) SNPs

**Fig 2:**
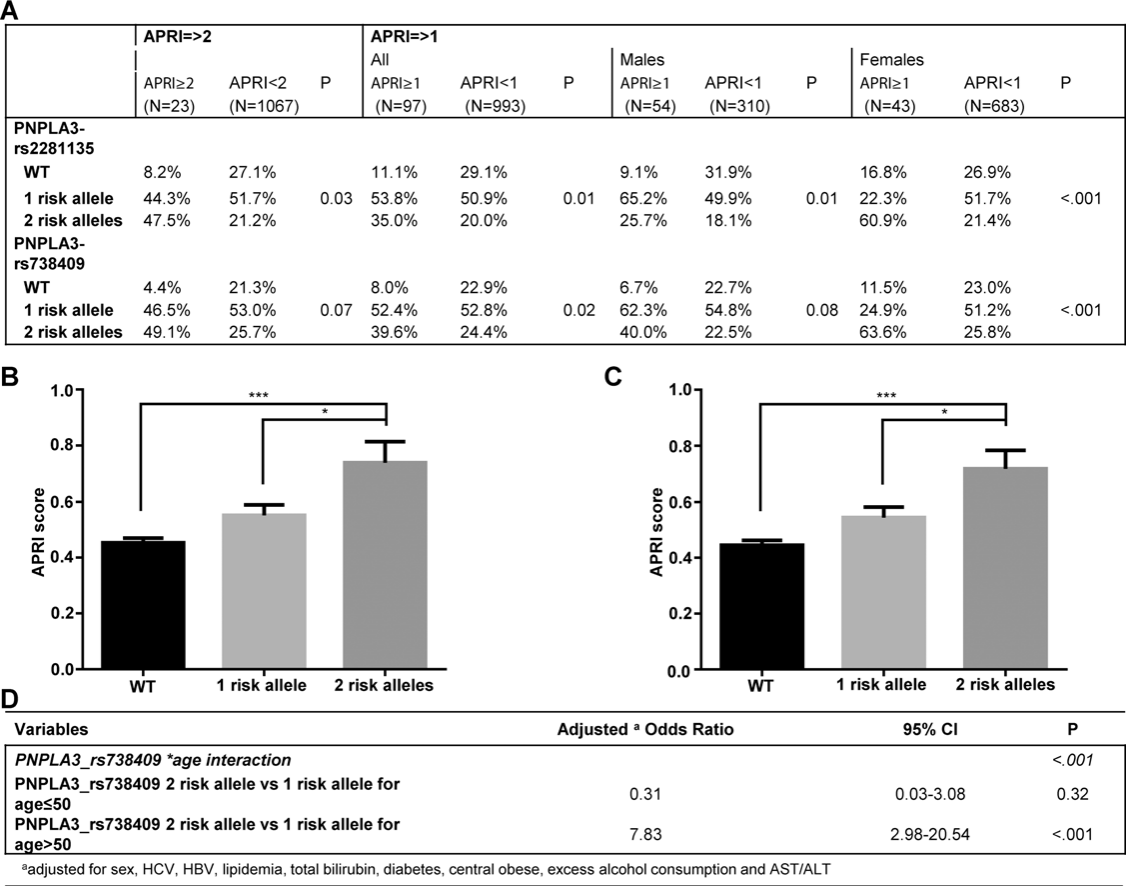
PNPLA3 SNPs and APRI scores. (**A**) PNPLA3 SNPs genotype distribution according to cirrhosis (APRI cutoff of 2) and cirrhosis/advanced fibrosis (APRI cutoff of 1) status. APRI scores in CCHC participants carrying PNPLA3 rs2281135 (**B**) and rs738409 (**C**) SNPs. (**D**) Association between PNPLA3 rs738409 and cirrhosis/advanced fibrosis (APRI cutoff of 1) based on multivariable logistic regression model. APRI = Aspartate transaminase to Platelet Ratio Index; SNP = single-nucleotide polymorphisms; PNPLA3 = patatin-like phospholipase domain-containing-3; CCHC = Cameron County Hispanic Cohort.

Since PNPLA3 SNP rs738409 has been reported to be associated with liver fibrosis and PNPLA3 SNPs rs738409 and rs2281135 have been associated with elevated transaminases in CCHC participants, correlation analysis between these two SNPs and APRI scores was performed. Overall, 47.5% of the participants with predicted cirrhosis carried 2 risk alleles of PNPLA3 SNP rs2281135 compared to 21.2% of the non-cirrhotic participants (p = 0.03). Similarly, 49.1% of the participants with predicted cirrhosis carried 2 risk alleles of PNPLA3 SNP rs738409 compared to 25.7% in non-cirrhotic group, although the difference did not reach significance (Fig 2A). There were significantly more participants in the cirrhosis/advanced fibrosis group who carried 2 risk alleles of both SNPs compared to participants with no predicted advanced fibrosis (35.0% vs 20.0% for SNP rs2281135; p = 0.01 and 39.6% vs 24.4% for SNP rs738409; p = 0.02) (Fig 2A). Participants with 2 PNPLA3 rs2281135 risk alleles had significantly higher APRI scores (0.74±0.07) compared to participants carrying no (0.45±0.02) or 1 (0.55±0.04) risk allele (p < .001 and p = 0.01, respectively) (Fig 2B). Similarly, participants with 2 PNPLA3 rs738409 risk alleles had significantly higher APRI scores (0.72±0.06) compared to participants carrying no (0.44±0.02) or 1 (0.54±0.04) risk allele (p < .001 and p = 0.01, respectively (Fig 2C). Age was found to be a significant effect modifier of the relationship between rs738409 and cirrhosis/advanced fibrosis status (p < .001). Odds of cirrhosis/advanced fibrosis status for 2 risk alleles were significantly higher than those for 1 risk allele in participants >50 years old (adjusted OR = 7.83) based on the multivariable logistic regression model (Fig 2D).

### Risk Factor Synergy in Participants with Cirrhosis/Advanced Fibrosis

Finally, we characterized the presence of the identified risk factors (diabetes, central obesity, hepatitis C, excess alcohol consumption, and 2 PNPLA3 risk alleles) in each of the individuals that had APRI≥1. While at least one of these risk factors was present in all females, none were identified in 13% of males. These males were young with a median age of 32 years. The most common single risk factor observed in individuals with cirrhosis/advanced fibrosis was central obesity accounting for 13% of males and 11.6% in females. These individuals with central obesity as the single observed risk factor were older with a median age of 54.5 years (Table 4). The combination of central obesity and diabetes was observed in 21.4% of males and in 30.2% of females. Interestingly, the combination of central obesity and excess alcohol consumption in males led to the development of cirrhosis/advanced fibrosis at a significantly younger age (median 30 years) compared to central obesity alone (p = 0.02) or central obesity and diabetes combined (p = 0.01) (Table 4). In females, an additive effect between central obesity and 2 PNPLA3 risk alleles was observed with 51.2% showing both risks regardless of the presence of diabetes (Table 4).

**Table 4:** Percentage and median age of participants with cirrhosis/advanced fibrosis, with central obesity (CO) alone or in combination with diabetes (DM), excess alcohol consumption (Alc) or 2 risk alleles of PNPLA3 rs738409 (SNP) in males and in females.

**A Males**			
**Risk Factors**	**%**	**Median Age**	***P***
CO	13.0	43	0.02^b^
CO+DM	21.4	54	0.009^c^
CO+Alc^a^	12.5	30	
**B Females**			
**Risk Factors**	**%**	**Median Age**	
CO	11.6	66	
CO+DM	30.2	53	
CO+SNP^d^	27.9	42.5	
CO+SNP+DM	23.3	51	

CO, central obesity; DM, diabetes mellitus; SNP, single nucleotide polymorphism.

^a^ excess alcohol consumption

^b^p value between CO and CO+Alc

^c^p value between CO+DM and CO+Alc

^d^ 2 risk alleles of PNPLA3 rs738409

## Discussion

This is the first study to estimate the prevalence of cirrhosis in Hispanic adults in the US, focusing here on Hispanics in South Texas. To that end, we investigated a community-based Hispanic cohort in Cameron County. First, we applied a strategy recently employed to estimate the prevalence of cirrhosis in the general US population, using a non-invasive scoring system (APRI ≥2) [6]. In addition to this direct comparison to national data, we also used a APRI ≥1 to enhance sensitivity for detecting cirrhosis, supported by the findings of two large meta-analyses [31, 32].

Overall, we observed a 4-fold higher prevalence of cirrhosis in Hispanics in South Texas compared to the general US population. Several features of liver cirrhosis specific to this population compared to the overall US population were identified. The national prevalence steadily increased with age and peaked at 0.57% in those between 45 and 54 years old [6].Similarly, higher prevalence (1.28%) was observed in the 45–54 age group in our study group. However, in contrast to the national study, the highest prevalence (1.92%) was observed in the 25–34 age group. This high prevalence in the young age group was mostly contributed by males. In addition, while 72.7% of participants with predicted cirrhosis in the US were males, Hispanic females in South Texas were significantly affected Similarly, while viral hepatitis accounts for 46.7% of cirrhosis in the US population [6], it accounts for only 13.6% of cirrhosis among Hispanics in South Texas. Finally, a strikingly high prevalence of cirrhosis was observed in young Hispanics males (3.64% in 25–34 age group) while older age is an independent risk factor for cirrhosis in the national study. The early onset of cirrhosis in Hispanics is consistent with a previous epidemiological report [8]. Development of cirrhosis in patients under age 35 has been suggested to be related to adolescent onset of alcohol and parenteral heroin abuse [33]. The status of intravenous drug use and alcohol intake in adolescents in this cohort are currently unknown. Hispanics are the ethnic group with the highest prevalence of NAFLD in children and adolescents [34] and cases of children with NAFLD who develop cirrhosis in young adulthood have been reported [35]. While the prevalence of pediatric NAFLD in this population is currently unknown, a study performed on 325 high school students in Cameron County showed that 40% of these adolescents had high waist circumference and 27% exhibited insulin resistance [36].

A main finding of our study is the major contribution of central obesity to cirrhosis/advanced fibrosis in Hispanics in South Texas. While an association between central obesity and fibrosis progression in the setting of NAFLD has been previously suggested [37, 38], this is the first report identifying central obesity as the most frequent risk factor associated with cirrhosis/advanced fibrosis in a population. Remarkably, males with both central obesity and excess alcohol consumption presented with cirrhosis/advanced fibrosis at a significantly younger age (median age: 30) than males with central obesity alone or with central obesity and diabetes, suggesting for the first time a synergistic effect between central obesity and excess alcohol consumption. For 13% of males with predicted advanced fibrosis, no known risk factor was identified. These participants were also young (median age: 32). We can speculate that alcohol consumption was underestimated in this group and that these participants may have a history of central obesity in adolescence or earlier.

Genetic factors are involved in the development of liver cirrhosis [39] and we found a correlation between increased APRI scores and 2 risk alleles of PNPLA3 SNPs in the CCHC. Based on multivariable analysis, the carrier of 2 risk alleles of these SNPs was identified as an independent risk factor associated with cirrhosis/advanced fibrosis. Our findings are consistent with a study by Krawczyk et al. in a cohort of 899 European patients with chronic liver diseases, where homo-and heterozygous carriers of the G allele of rs738409 were at increased risk for developing cirrhosis and advanced fibrosis [40]. Besides PNPLA3, other genetic factors are involved in cirrhosis or fibrosis progression. In NAFLD patients, carriers of the minor allele of non-synonymous SNP rs58542926 in transmembrane 6 superfamily member 2 (TM6SF2) were found to have an increased prevalence of advanced fibrosis [41]. Moreover, a cirrhosis risk score consisting of SNPs in 7 genes (AP3S2, AQP2, AZIN1, DEGS1, STXBP5L, TLR4, and TRPM5) has been shown to predict fibrosis progression in HCV infected patients [42, 43]. Whether additional genetic factors influence the risk of fibrosis in the CCHC remains an area for future study.

This study has several limitations. The identification of cohort subjects with advanced fibrosis or cirrhosis is based on using a non-invasive marker APRI that has not been validated in population cohorts. APRI has been primarily validated among patients with chronic liver disease and there can be reasons for false-positive APRI elevations in a population-based sample including drug induced liver injury, acute alcoholic hepatitis or non-cirrhosis related thrombocytopenia. In addition, HCV and HBV were tested in 50% of the cohort participants. The missing data could therefore affect the PAF analysis when estimating the contribution of these factors to liver cirrhosis or cirrhosis/advanced fibrosis.

Liver cancer is the fastest growing cause of cancer-related mortality in the United States. Liver cirrhosis is the most common risk factor for hepatocellular carcinoma (HCC) and therefore the risk factors for HCC and cirrhosis largely overlap. These include genetic factors such as PNPLA3. PNPLA3 rs738409 2 risk alleles has been identified a risk factor for developing HCC associated with NAFLD [44], alcoholic liver disease [45] or chronic HCV infection [46, 47].The role of central obesity as a risk for HCC is also emerging as shown in a large European study [48]. The high prevalence of cirrhosis in Hispanics in South Texas seems to correlate with the high prevalence of HCC in this population. Recent reports showed that the incidence of HCC is the highest among Hispanics in South Texas [49, 50]. In Cameron County, liver cancer ranked third among cancers in males and sixth in females based on self-reported data [51]. Because the survival rate of HCC is dismal, it is critical to identify those at high risk and enroll them in surveillance and prevention programs. Our study can therefore contribute to that effort. Affordable non-invasive diagnostic means to detect and stage liver fibrosis are urgently needed for this underserved population.

## Conclusions

We found an overall prevalence of 0.94% for cirrhosis and of 3.54% for cirrhosis/advanced fibrosis in the CCHC. This high prevalence of cirrhosis and advanced fibrosis in South Texas affects in particular young Hispanic men. The majority of cirrhosis and advanced fibrosis risk was contributed by central obesity and diabetes. Synergistic effects were also observed between central obesity and excess alcohol consumption. These findings are an important first step in developing interventions to reduce liver disease burden in this underserved population. Public health efforts are needed to understand the unique pattern of cirrhosis and advanced fibrosis in Hispanics in South Texas, to reduce this disease burden and to identify those to whom HCC surveillance may be targeted.
